# How can early life adversity still exert an effect decades later? A question of timing, tissues and mechanisms

**DOI:** 10.3389/fimmu.2023.1215544

**Published:** 2023-06-30

**Authors:** Archibold Mposhi, Jonathan D. Turner

**Affiliations:** Immune Endocrine and Epigenetics Research Group, Department of Infection and Immunity, Luxembourg Institute of Health (LIH), Esch-sur-Alzette, Luxembourg

**Keywords:** stress, early life adversity, stem cells, progenitor cells, metabolism, mitochondria

## Abstract

Exposure to any number of stressors during the first 1000 days from conception to age 2 years is important in shaping an individual’s life trajectory of health and disease. Despite the expanding range of stressors as well as later-life phenotypes and outcomes, the underlying molecular mechanisms remain unclear. Our previous data strongly suggests that early-life exposure to a stressor reduces the capacity of the immune system to generate subsequent generations of naïve cells, while others have shown that, early life stress impairs the capacity of neuronal stem cells to proliferate as they age. This leads us to the “stem cell hypothesis” whereby exposure to adversity during a sensitive period acts through a common mechanism in all the cell types by programming the tissue resident progenitor cells. Furthermore, we review the mechanistic differences observed in fully differentiated cells and suggest that early life adversity (ELA) may alter mitochondria in stem cells. This may consequently alter the destiny of these cells, producing the lifelong “supply” of functionally altered fully differentiated cells.

## 1 Introduction

Since the early work of David Barker linking birthweight to later life cardiovascular disease (1) the link between many different forms of environmental exposure in the first 1000 days has been expanded to many psychopathologies [reviewed in (2)], as well as long-term immune-mediated diseases (3), metabolic diseases such as diabetes and obesity (4), and epidemiological studies suggest that such early-life adversity (ELA) is also negatively associated with life expectancy (5). Stress itself impacts many physiological systems and for many years there has been a focus on the brain as it controls the two principal stress-response pathways, the autonomous nervous system (ANS) and hypothalamus-pituitary-adrenal axis (HPA) (6). There is a general consensus that exposure to stress or adversity in early life increases the risk of developing disorders that persist throughout life, as in the original paradigm of Barker et al. (1, 7, 8). This early-life period is a particularly sensitive developmental window in which many biological systems are not only established, but their settings are adapted to the local environment (9).

**Figure 1:**
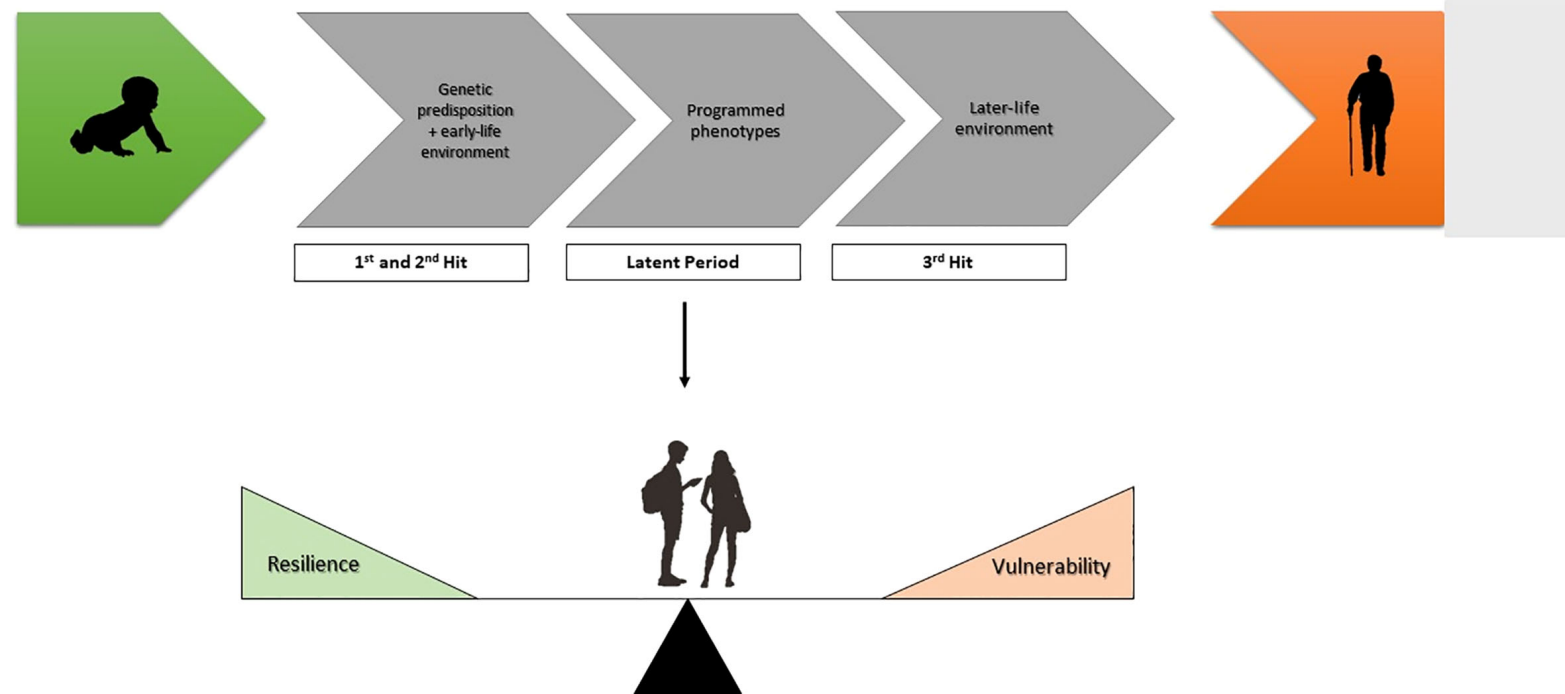
The three-hit model conceptualising early life adversity (ELA). Genetic predisposition and experience-related factors in the early-life environment comprise the first and second hit respectively. After the early-life exposure phase there is a latent phase, whereby phenotypes become programmed. The development of disease depends on the susceptibility or resilience of these programmed phenotypes to later-life challenges (third hit).

This sensitive window is a key element of the current “three-hit” model of the Developmental Origins of Health and Disease. This was initially proposed by Daskalakis et al. in 2013 (10). This takes the “fixed” genome as an immutable first “hit” that is then present lifelong, providing a baseline genetic risk for disease. This is accompanied by a second “hit” based on the environment that the individual is exposed to during such critical developmental windows. Again, this second “hit” is not sufficient to induce disease alone. These are then combined into a latent of “susceptible” phenotype that requires a subsequent element, a third “hit”, many years later for the disease risk to be crystallized (**Figure 1**). The absence of any one “hit” results in a healthy individual and the risk associated with either the genetic or exposure risk never crystallises. This was initially conceptualized as a cumulative model where stressors were thought to accumulate throughout life until a critical threshold was passed and (psycho)pathology emerged (11), however, the “three-hit model” suggests that the environment during the sensitive developmental window is part of a “predictive adaptive response” (12). A corollary to this is that if the body of the individual adapts to the anticipated environment throughout life, the third hit may be the mismatch between the anticipated later-life environment and the actual environment encountered, called the match/mismatch hypothesis. Similarly, the “three hit” model places a genetic susceptibility in an environmental context. As originally hypothesised by Belskey and Beaver “a genetic vulnerability in one environment may actually constitute an adaptive benefit in another environment” (13), putting a theoretical basis under the gene-environment interactions that are regularly reported.

Evidence for this sensitive developmental period come from a number of natural experiments as well as *in vivo* models. There is now very strong evidence that short stress exposures lasting days or weeks, rather than months is a sufficient “second hit” to induce a significant disease risk. One of the classic natural experiments was the 1998 Quebec ice storm (14). The Quebec Ice Storm was a particularly harsh but localised meteorological event, where that the local population was deprived of electricity and all major facilities for between two weeks to two months (15). Twenty years later children exposed to the ice storm either *in utero* or immediately post-partum had altered metabolic parameters (BMI, insulin resistance) and increased HPA axis reactivity (16, 17). In a similar manner, the Dutch Hunger Winter, a consequence of the German-imposed food embargo in 1943-44 in the Netherlands demonstrated the importance of the timing of the stress exposure (18, 19), although, in this cohort, lifelong mental health problems were observed in addition to the altered metabolic phenotype (20, 21), again mediated by changes DNA methylation (22).

Furthermore, the mother-infant bond is established in the immediate post-partum period, and any negative psychological or psychosocial event will alter this bond (23, 24). This concords with research from the institutionalisation-adoption paradigm where the carer-infant bond has been shown to be primordial in setting lifelong health trajectories, and that the psychosocial element is the strongest (25). Unstable care in an otherwise materially adequate environment negatively impacted physical, neurological, and cognitive development; attention, emotion and behaviour; stress-axis functioning and immune system functioning many years later (26). Our work on this paradigm shows that even the shortest time windows for adoption that have been studied so far (around 2 months) left a strong immune, behavioural, and health phenotype 24 years later (27–31)

Despite the progress made over the past decades, there are many unanswered and emerging questions. First of all, as outlined below, we are currently concentrating our research efforts on somatic, fully differentiated cells, however, are these the genuine target of the early-life programming rather than a modification of the developmental trajectory? Furthermore, as there appears to be an early life window of susceptibility that leads to a life-long production of programmed somatic cells are we looking at a process in which undifferentiated stem cells are programmed to produce a lifelong supply of modified somatic cells? And finally, are we looking at a ubiquitous mechanism (or series of mechanisms) common to many types of stem cells or are there mechanisms specific to each cell type? This article attempts to address these questions, taking into account the current literature in light of these unanswered questions.

## 2 Hallmarks of embedding: the brain and the immune system

Extensive reading of the early-life adversity literature suggests that such exposure principally affects the central nervous system and the immune system. Long-term observational studies have associated ELA with an increased risk of developing stress-related disorders (32) mental health problems, as well as diabetes, obesity, asthma/allergy, cardiovascular disease and depression (33). Although the pathophysiology and aetiology underlying these associations remains obscure, there appears to be “an unholy trilogy” of interactions between the immune and central nervous system, partly through the hypothalamus-pituitary-adrenal stress axis [reviewed in (34)].

At birth, the development of the human brain is not complete, and it continues throughout the first years of life (35). This makes the brain particularly sensitive to the external environment during this early life period. ELA not only increases the risk of mental health problems such as schizophrenia and depression, but it also induces changes in white matter organization and grey matter volume as well as changing autonomic nervous system function and activity, HPA axis activity, as well as behaviours such as emotion and attention (36). For example, data from our laboratory suggests that the HPA axis is perturbed for up to 24 years after exposure to ELA (31, 37, 38), although peripherally, HPA axis derived glucocorticoid effects are functionally unaltered (29). Within this context, the role of the hippocampus is particularly important. The hippocampus is considered part of the approach-avoidance system (39), indirectly feeding inhibitory signals into the hypothalamus controlling the hormonal stress reaction (40), modulating basal glucocorticoid (GC) levels and the duration of HPA axis activation (41–43).

At the same time, ELA prematurely ages the immune system, and this has been extensively reviewed elsewhere (34). Both the innate and adaptive immune systems are clearly impacted. In the adaptive immune system CD8+ cytotoxic T cell are the most strongly affected (28) disturbing the CD4/CD8 balance in a similar manner to chronic viral infections (44–46), and showing signs of elevated stimulation (28) and accelerated ageing and senescence (28, 44). CD4+ T helper cells are not, however, unaffected, with an increase in senescent (CD57+) cells (44) and T_helper_17 cells (28). Interestingly, an overall lower percentage of B cells was associated with ELA in both human and animal studies (45, 47, 48), without clear causal explanation. In the innate immune system, there are clear differences in macrophages and natural killer (NK) cells. There reduced numbers, of macrophages and they have a lower proliferation, and migratory capacity (49) that is probably programmed by exposure to glucocorticoids during the early-life stress period (50). Similarly, ELA reduces the number of circulating NK cells, decreases their degranulation capacity, and pushes them to an aged, senescent phenotype earlier (51). Overall, these changes make the immune system significantly less capable of functional reactions (e.g. cytotoxicity, degranulation, proliferation) to pathogens, and more senescent and pro-inflammatory [reviewed in (34)]. The question remains as to how this occurs and whether this reflects a short-term immune advantage with a long-term cost. Exposure to latent regularly reactivating viruses such as CMV goes some way to explain the underlying mechanism (30, 44).

Changes to brain and immune system do not occur in splendid isolation; however, they are both an interdependent part of the overall ELA-phenotype. Rodent models such as maternal deprivation concurrently induce behavioural and hippocampal changes (52) as well changes in both the neuro- and peripheral immune systems (51, 52). Within the brain, maternal deprivation leads to neuroinflammation and it increases astrocyte and microglia numbers as well as the expression of inflammatory cytokines (53, 54), and this seems to be a more general phenomena as other tissue-resident sentinel cells such as macrophages are part of a bi-directional immune-central nervous system (CNS) crosstalk (55). As in the rat models, the human institutional adoption paradigm also induces concurrent changes in behaviour and the immune system with an increased risk of mental health problems (27–30). When the full picture was analysed, although from cohorts dedicated to studying the immune system or CNS independently, it appears that ELA plays a role amplifying the interaction between the peripheral immune system, in particular chronic low-grade inflammation, acts on both threat and reward circuits to accelerate the development of both physical and mental health problems [reviewed in (55)]. While the mechanism by which ELA excepts its effect concurrently on the immune and nervous system it is clear that the normal homeostasis of these systems is somehow perturbed, reaching a new, potentially pathological state together (34, 56).

Although we are comparing “head to head” changes in the immune system and the brain, it is worth remembering that these two systems interact. Pioneering work by the Jankovic laboratory throughout the 1980s and 90s established the field of neuroimmunomodulation (57). Innervation of immune tissues such as the spleen by SNS generates immune synapses where the environmental situation is passed on to both pro- and anti-inflammatory macrophages (58) affecting many other parameters as well (59). However, whilst these seminal studies have paved the way to the current investigation of how ELA influences immune, neuronal and metabolic changes in later, they are beyond the scope of this review.

## 3 Lifelong programming in the brain and immune system

To date, we have limited ourselves to differentiated cells in somatic tissues. Is it possible that we have been barking up the wrong tree? During the early stages of life, the brain and the immune system present a high degree of plasticity which makes them more sensitive to environmental stressors and may result in lifelong effects being visible decades after exposure (8, 9, 34). Exposure during the early-life period has to somehow become “embedded” in order to maintain a stable phenotype over many decades. This has been assumed to pass through stable epigenetic mechanisms such as DNA methylation (60). Over the past two decades there has been considerable effort in determining the epigenetic modifications, particularly in somatic cells and tissues such as buccal epithelial cells or circulating white blood cells (60). However, it has long been accepted, although rarely stated, that somatic cells have a finite life that is often orders of magnitude shorter than the duration of the early-life adversity-associated phenotype. There is, however, considerable heterogeneity in cellular lifespan (61). Tissues balance cellular death and proliferation to remain in homeostasis. There are tissues that are particularly long lived, e.g. heart muscle and brain, maintained by long lived slowly proliferating cells, whereas keratinocytes and immune cells have a very short lifespan and a high proliferation rate (61). With the exception of adipose tissue, heart muscle, neocortical neurons, smooth and skeletal muscles all other cell types live less than 1.5 years (61).

**Figure 2:**
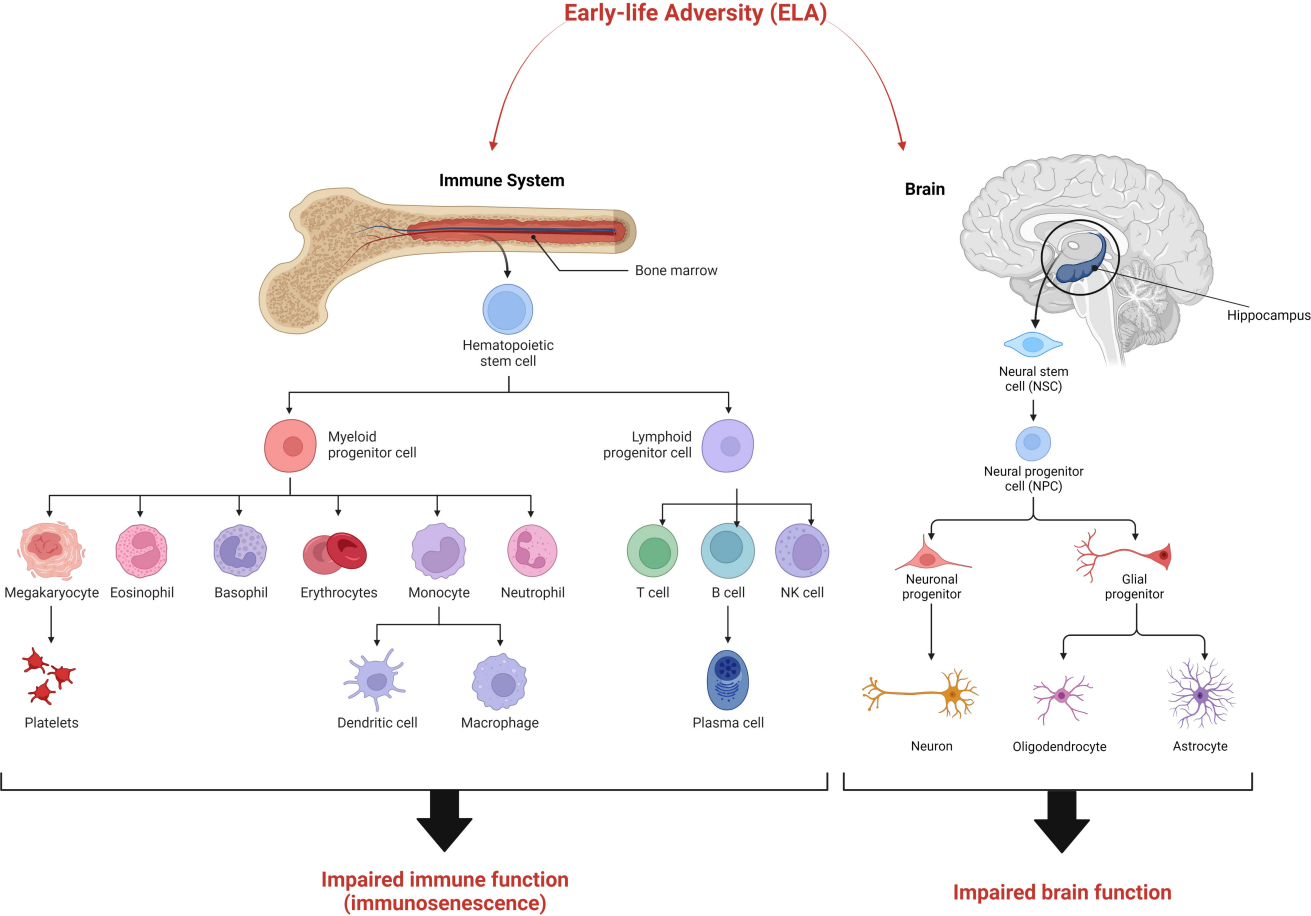
The “stem cell hypothesis” explaining the maintenance of the ELA phenotype over many decades. We suggest that early-life adversity exerts its effects on stem cells and/progenitor cells in the brain and immune system resulting in the development of aberrant differentiated cells with impaired function. The resulting ELA phenotype is characterised by immunosenescence and impaired cognitive function.

This “*elephant in the room*” has an obvious answer. Since terminally differentiated cells are replaced from partially differentiated progenitor cells, it is actually these progenitor or stem cells that are affected (**Figure 2**). Once programmed (or “embedded”) they continue to generate somatic cells that are different from those in unexposed individuals throughout the duration of the phenotype. Indeed, the “perinatal sensitive window” may be a period in which such progenitor cells are naturally susceptible to such programming in order for the body to adapt to its environment. This is supported by data from the two tissues that seem to be the most affected by exposure to early-life adversity, the brain, and the immune system.

In the context of psychosocial stress, the endocrine stress response is largely controlled by the hippocampal inputs into the HPA axis (42). The hippocampus is somewhat unique in that neurogenesis occurs here throughout life, and several thousand new-born dentate granule cells are generated and inserted into pre-existing neural circuits every day (62, 63) and are necessary for normal hippocampal function. These dentate gyrus cells are the product of hippocampal neurogenesis that is thought to be regulated by a number of metabolic factors (e.g. IGF-1 VEGF, and FGFys) as well as behavioural-stimuli released neurotransmitters such as GABA. Cerebral neurogenesis is not limited to the hippocampus, as stem cells have also been identified within both the striatum (64) as well as the dorsolateral region of the lateral ventricule (65). In both cases, these stem cells could differentiate into glia and neuronal cells (64, 65). There is also evidence to suggest that early stress impairs their ability to proliferate and differentiate [reviewed in (2)]. Many common early-life adversity paradigms such as Maternal Separation, Limited Bedding, or exposure to maternal immune activation *in utero* induce major cognitive impairments later in life that are associated with this reduced repair capacity [reviewed in (2)].

According to a recent study in mice, it was observed that early life stress impairs cell proliferation in the hypothalamic parenchyma and reduces the number of putative hypothalamic neural stem cells at 4 months (66). In another study, exposure to early life stress impaired hippocampal development in mice by promoting the depletion of adult neural stem cells in the dentate gyrus (67). To our knowledge, no further study has been carried out to determine the mechanisms underlying this impaired proliferation of neural stem cells. It is plausible that the number of subsequent neural lineages are affected by this decrease in the stem cell population which has far reaching consequences on brain development. This might be another treasure trough linking early life adversity to the development of cognitive disorders in humans.

There are several indications in literature that hippocampal neurogenesis is an adaptive response to environmental and/or internal challenges (68). We hypothesize that upon exposure to adversity or stress during early life, undifferentiated stem/progenitor cells are programmed to produce a lifelong supply of aberrant somatic cells. Consequently, by focussing on differentiated cells in the brain we are merely observing distal aberrations which have cascaded from neural stem/progenitor cells over time. It is therefore, important to broaden our scope to include stem/progenitor cells since they are the source of anomalous somatic cells.

In a similar manner to the hippocampus, almost all types of circulating blood cells are permanently being generated and released from the bone marrow, where hematopoietic stem cells (HSC) reside (69). Many immune cell populations are very short-lived such as dendritic cells, monocytes and granulocytes (70). Although T and B cells are longer lived and are partly maintained through their normal proliferative actions, these populations are also continually replenished with new-born naïve cells effectively maintaining polyclonality in their specificities (71). The only exceptions to this are cells such as tissue resident macrophages and B1-a innate-like lymphocytes that develop during embryogenesis (72).

Many studies by both ourselves and others, have shown that ELA is associated with changes in immune cell function. Here, we propose that exploring epigenetic changes in the haematopoietic stem cell population during the early stages of life post exposure to psychosocial stress would provide more information on the lifelong effects of ELA in the development of disorders of the immune system. Previously, in a study in mice it was shown that social stress promotes the migration of hematopoietic stem progenitor cells (HSPCs) from the bone marrow to the spleen where they actively proliferate and differentiate to produce monocytes, neutrophils and erythrocytes. No further studies have been carried out to test immune cell functionality in these cell lineages. In our previous study we have identified impaired NK cell function in a rat ELA model. In this regard it would be interesting to extend our analysis to all HSPCs derived immune cells.

It is worth mentioning that stem cells and their early descendants, progenitor cells, possess two unique traits, the ability to almost indefinitely proliferate in their undifferentiated state and secondly, their ability to differentiate into the ~200 distinct human cell types (73). As differentiated cells have a finite lifespan, there is a continuous turnover with loss and replacement of these cells from the pool of dividing stem cells, differentiating into the cells required (74). It would appear logical to conclude that exposure to an environment that affects such stem or progenitor cells will have long-term consequences. Epigenetic programming of stem, rather than differentiated cells will have a persistent effect, generating epigenetically programmed stem/progenitor cells as they proliferate, as well as epigenetically programmed cells as they differentiate. Consequently, their epigenetic programming will have a far greater impact and potential for causing disease than that of the differentiated cells that are currently the focus of our attention. This “stem cell hypothesis” goes a long way to explain how ELA leaves a lifelong imprint that can have functional and pathological effects many decades later.

## 4 Mechanisms

Although detailed cellular and molecular mechanisms by which early life adversity induces long term effects have not been described, there is a large amount of circumstantial evidence that guides us towards several possible underlying mechanisms. Using the two most studied tissues, brain and peripheral blood cells, there are mechanisms that appear to apply to one system, the other, or potentially both. In light of the previous section, if it is the stem/progenitor cells that are actually programmed, here we outline the end result of that programming. The obvious corollary to the “stem cell hypothesis” is that the underlying mechanism that leads to the production of functionally divergent differentiated cells has yet to be examined.

### 4.1 Regulation of gene transcription

Since the seminal work of Weaver and Meaney in the early 2000s it has been clear that changes in the overall epigenetic landscape, DNA methylation, and gene transcription contribute towards maintaining the ELA phenotype (75–78). DNA methylation changes have been reported in many tissues including blood, and the brain, particularly in the hippocampus and prefrontal cortex (PFC) (79–83). And importantly, recent work suggests that ELA-induced epigenetic changes, as well as their downstream transcriptional effects are sex-dependent (84). Although a wide number of genes have been identified and investigated, there seems to be a central dyad of the glucocorticoid receptor (GR) and Brain Derived Neurotrophic Factor (BDNF).

#### 4.1.1 ELA and glucocorticoid receptor methylation

Epigenetic regulation of the GR has been extensively discussed elsewhere, however, the GR gene (NR3C1 in man, Nr3c1 in rodents) structure makes it highly amenable to epigenetic regulation (85–90). The environmentally regulated, complex, 5’ untranslated region controls tissue-specific GR expression, GR protein isoform production and cell surface trafficking (90–92). Initial work in rodents suggested that maternal care (LGABN; Licking, grooming and arched-back nursing) modified Nr3c1 methylation in both the cerebellum and the hippocampus (93, 94). This was reported to affect transcription factor binding, reducing Nr3c1 transcription (85, 89, 95) and reduced Gr levels are associated with increased glucocorticoid responses to stressors (reviewed in (96, 97). The situation is, unfortunately, less clear in humans. A range of strong pre-natal adversities including anxiety, depression, or intimate partner violence, have all been linked to increased levels of NR3C1 methylation peripherally (98–101). More recently, the effect of exposure to the COVID-19 pandemic in different trimesters of pregnancy suggests that lockdown (and associated stress) during the first trimester induced lower NR3C1 methylation levels than exposure during the second or third trimesters (102). Similarly, post-natal stress exposure (“childhood maltreatment, parental loss, and low levels of parental care”) has been linked to increased NR3C1 methylation later in life as both pre-schoolers, teenagers, and later on as adults (103–105). However, in the only study examining NR3C1 methylation, expression and function, ELA exposure did not affect methylation levels, and the receptor remained functionally identical (29).

The hypothesis of NR3C1 methylation linking ELA to later life psychopathology is well established [reviewed in (106) and (97)], and an association has been observed in adults with i) borderline personality disorder (107), ii) both bulimia and borderline personality disorder (108), iii) internalizing behaviour problems, iv) arousal and excitability. Interpretation, however, is made more complicated due to the limited overlap of CpGs investigated between different studies, the broad variety of stressors investigated and the overall complexity of the GR CpG island regulatory region (29, 90). Furthermore, there is doubt as to the clinical relevance of the small methylation changes being reported for the GR and the absence of a clear, direct, causal link between NR3C1 methylation and eventual transcript and protein levels (92, 109).

The hypothesis of epigenetic GR regulation remains appealing since a small number of longitudinal studies have demonstrated changes in NR3C1 and BDNF promoter methylation over the life course (110), even if in our study, 24 years post adversity limited (or no) methylation differences were observed (29).

#### 4.1.2 ELA and BDNF methylation

As for the GR, methylation of the brain-derived neurotrophic factor (BDNF) gene promoter is clearly influenced by the early life environment in both the brain and immune cells with clear effects on BDNF protein levels (111). Unlike the GR which controls HPA axis reactivity lifelong, BDNF is involved in brain development as well as subsequent synaptic transmission and neuroplasticity. Changes in BDNF gene expression levels peripherally and centrally have been associated with mental health problems commonly associated with ELA exposure (111). While the overall effect of altered GR and BDNF gene methylation may be different, their mechanisms converge on mitochondrial processes (112). GR proteins are traditionally cytosolic, with a small percentage being trafficked to the outer cell membrane (92), however, they are also translocated into mitochondria. Here, GR regulates bio-energetic processes including oxidative phosphorylation, while BDNF, helps control the biogenesis and transport of mitochondria (2).

#### 4.1.3 BDNF and GR transcription effects influence mitochondria

Mitochondria are a key component in regulating intracellular stress response and they harbour a complex signalling network which enables cells to sense internal or environmental changes and adjust in response to these stimuli (113). From previous studies it is evident that environmental factors also promote epigenetic changes which negatively impact the mitochondria’s adaptive response to stress (113–115). Mitochondria orchestrate stress-signalling pathways within the endocrine, immune and central nervous system (CNS) by adjusting their activity to suit the prevailing energetic demands (116). Perturbed BDNF-GR signalling can promote mitochondrial dysfunction by disrupting essential bio-energetic and transport processes which can potentially result in the development of metabolic and cognitive disorders associated with the neuroendocrine and immune systems (112).

**Figure 3:**
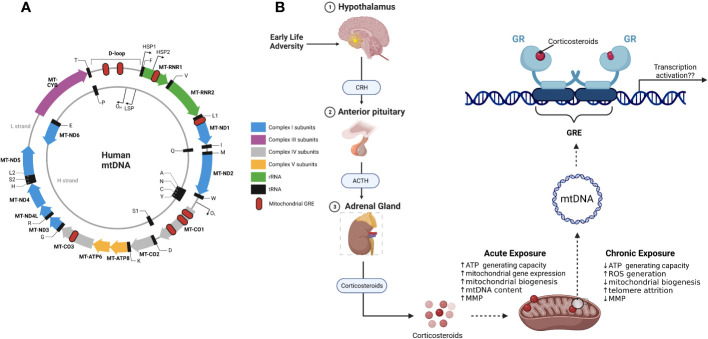
Mitochondrial DNA and mitochondrial glucocorticoid receptors. **(A)** schematic representation of the mitochondrial genome with mitochondrial genes (solid coloured arrows), transcription start sites (outward thin black arrows) and the 8 putative glucocorticoid response elements (GRE; red oval); **(B)** Effects of ELA on the HPA axis results in the release of corticosteroids. GR binds to the mitochondrial DNA in the presence of corticosteroids. Depending on the length of exposure to corticosteroids, GR modulates mitochondrial gene expression and consequently mitochondrial function. CRH, Corticotropin releasing hormone; ACTH, Adrenocorticotropin hormone; GR, glucocorticoid receptor.

Under conditions of stress or in the presence of elevated levels of corticosteroids, GR translocates to the mitochondria and there it binds to the D-loop regulatory region of the mitochondrial DNA (mtDNA) thus modulating the expression of mitochondrial genes (**Figure 3**) (117). In addition to the D-loop, other GR binding sites (Glucocorticoid Response Elements, GREs) have been discovered in mtDNA and these give support to the hypothesis that mitochondrial GR directly influences mitochondrial gene transcription and overall mitochondrial physiology (118, 119). Mitochondrial gene expression is directly linked to mitochondrial function and therefore any factor that has the potential to regulate the expression of mitochondrial genes can influence mitochondrial activity (120). MtDNA methylation in the early life adversity context is an interesting avenue to explore given recent studies that have shown that methylation of the mtDNA promotes mitochondrial dysfunction (121, 122). In nuclear DNA, methylation within promoter regions affects the binding of transcription factors and subsequently leads to altered gene expression. Whether a similar gene expression regulation mechanism also exists for modulation of mitochondrial genes remains largely unexplored. In this regard, it would be interesting to determine whether the methylation status of the mitochondrial genome at regulatory regions such as the D-loop has an effect on the binding of transcription factors such as GR during stress conditions.

BDNF is known to promote mitochondrial function *via* activation of its receptor, tropomyosin receptor kinase B (TrkB), which stimulates peroxisome proliferator-activated receptor gamma coactivator 1-alpha (PGC1α) mediated mitochondrial biogenesis in neurones (123). This suggests that decreasing BDNF expression can potentially affect mitochondrial abundance and therefore attenuate adaptive response to stress. According to various studies, it is circumstantially evident that BDNF and GR expression affects mitochondrial functioning during early life adversity when an individual is subjected to psychological stress (2).

### 4.2 Telomeres, DNA repair and genomic stability

There is an intimate symbiotic interrelationship between DNA repair and telomere maintenance mechanisms that maintains genome stability and integrity. Telomeres, conserved DNA repeated sequences, cap the ends of eukaryotic chromosomes preventing their recognition as “damaged DNA” ensuring chromosome integrity and genome stability during cellular replication (124). DNA repair covers a series of mechanisms that identify and repair damaged DNA including i) base excision repair, ii) nucleotide excision repair, iii) mismatch repair, and iv) homologous recombination (125). Consequently, any disturbance in these signalling pathways results in genomic instability and potentially disease. Crosstalk between the DNA repair and telomere maintenance mechanisms means that when telomerase levels or activity is reduced so too is the DNA damage repair response (126) and vice versa (127). In the context of early life adversity, there are a number of studies, particularly from the Entringer/Wadhwa laboratories that have demonstrated long-term stress-induced changes in the telomere/telomerase system (128, 129). There is no clear link so far between ELA and DNA integrity, however, some older data suggests that it is linked in a reversible manner to psychosocial and traumatic stressors later in life (130). This is, most probably, an indirect effect since activation of the GR by cortisol reduces transcription of important genes within the DNA damage repair system (129).

The basic principles of telomere biology have been extensively reviewed elsewhere (124). Briefly, the telomere end caps lose a few base pairs with each cell division and these are replaced by telomerase. However, telomerase has many additional functions [reviewed in (131)]. During embryogenesis telomerase modulates multiple cellular signalling pathways. Later in life, telomerase plays a significant role in activating resting stem cells into a proliferative state as well as maintaining their proliferative capacity and survival under physiological stress. However, unlike somatic cells, which become either senescent, apoptotic or autophagic, it has been shown that murine stem cells with shortened telomeres maintain their ability to proliferate but they lose the ability to stably differentiate into respective cell lineages (132, 133). According to our proposed hypothesis, exposure to ELA results in long-term stress-induced changes in the telomere/telomerase system, which impairs stem cell differentiation into functional somatic cells.

The general decline in telomerase activity during cellular differentiation is also important to take note of because telomerase has crucial extra-nuclear functions, particularly in the mitochondria. Here, it regulates both the mitochondrial membrane potential and superoxide production (134) processes that are themselves important regulators of mitochondrial efficiency, energy production, and oxidative stress. Diminished telomerase activity, as observed in a murine model, results in mitochondrial dysfunction characterised by high levels of reactive oxygen species (ROS) (135). Evidently, this sets into motion a vicious cascade of events where elevated mitochondrial ROS levels promote DNA damage, which consequently results in senescence. Overall, these events are of particular interest in the observed ELA immunophenotype and may be pivotal in determining the fate of stem cells (also see section 4.4.2).

Both DNA repair and the telomere system may also be important in the context of ELA as they both appear to be very plastic during this period. Inter-uterine conditions, e.g. gestational diabetes, inflammation, maternal psychological state, determine telomere length at birth (128, 129). Similarly, maternal exposure to air pollution (including smoking), gestational diabetes, and pre-term-birth/low birth weight increase DNA damage, and reduce DNA repair capacity (136, 137)

### 4.3 Impaired autophagy and proteostasis

ELA has become an established risk factor for neurodegenerative diseases, with standard models of adversity such as maternal separation, maternal immune activation and limited bedding and nesting (LBN) (2). In addition to operating at the (epi) genomic level, translational and protein stability mechanisms have also been proposed. The maintenance of adequate functional protein levels, proteostasis, incorporates the synthesis, folding, stability and eventual degradation of the proteins within the proteome (reviewed in (2)). This is disturbed in early life models such as maternal separation (MS), and is thought to lead to an abnormal “unfolded protein response” (UPR (138):) that potentially impairs autophagy, providing an environment in which age-associated neuropathies start to develop (139). MS-induced changes in autophagy and proteostasis are visible in the rat hippocampus at 3 months, persisting to 16 months. This is, however, a tissue-specific reaction, limited to the hippocampus and surrounding cortical areas appear to remain unaffected (140). Furthermore, like many other early-life adversity effects, this appears to also involve the mitochondrial unfolded protein response in both the hypothalamus and the hippocampus disrupting healthy brain aging after exposure to metabolic stimuli as well (2). The LBN model is, in many ways an experimental proof of the 3-hit hypothesis. Using biAT mice a bi-genetic model of Alzheimer’s disease, exposure to an additional early life stressor, in this case a dearth of suitable bedding and nesting material during the first days of life, significantly reduced long-term survival, with symptoms starting earlier, progressing faster, and reaching humane endpoints earlier, whilst the phenotype was rescued by an enriched and somewhat ‘positive’ early environment (2, 141). Similarly, in other genetic models of Alzheimer’s disease exposure to maternal separation of limited bedding and nursing increases the rate of cognitive decline, increased plaque deposition and reduced life expectancy (2, 141). There is no evidence so far which shows that autophagy and proteostasis are affected in stem cells after exposure to ELA. However, the autophagy network in adult neural stem cells regulates proteostasis and is essential for maintaining the pool of stem cells for life long neurogenesis (142). It is interesting to hypotheses that the impaired autophagy seen in the LBN model might also be applicable within the context of our stem cell model.

### 4.4 Metabolic regulation

From our previous work, it has become clear that not all immune cell populations are equally affected. However, there are many common points between those cell types that are affected, with a clear bias towards the induction of an inflammatory senescent phenotype (28, 30, 51). This is accompanied by a biasing of macrophages towards an M1 rather than an M2 phenotype as well as inducing an inflammatory rather than immunosuppressive phenotype (143). As we have previously shown, it is unlikely that epigenetic, transcriptional or functional changes in the GR underlie the immunophenotype, despite the strong immunomodulatory effect of glucocorticoids such as cortisol (29). There was no evidence of dysregulation in either GR signalling or function in monocytes as well as T and B cells lymphocytes (29). Our current interpretation is that the induction of a senescent immunophenotype with the accumulation of CD57+ terminally differentiated T cells and activated NK cells implies differences in immunometabolism.

**Figure 4:**
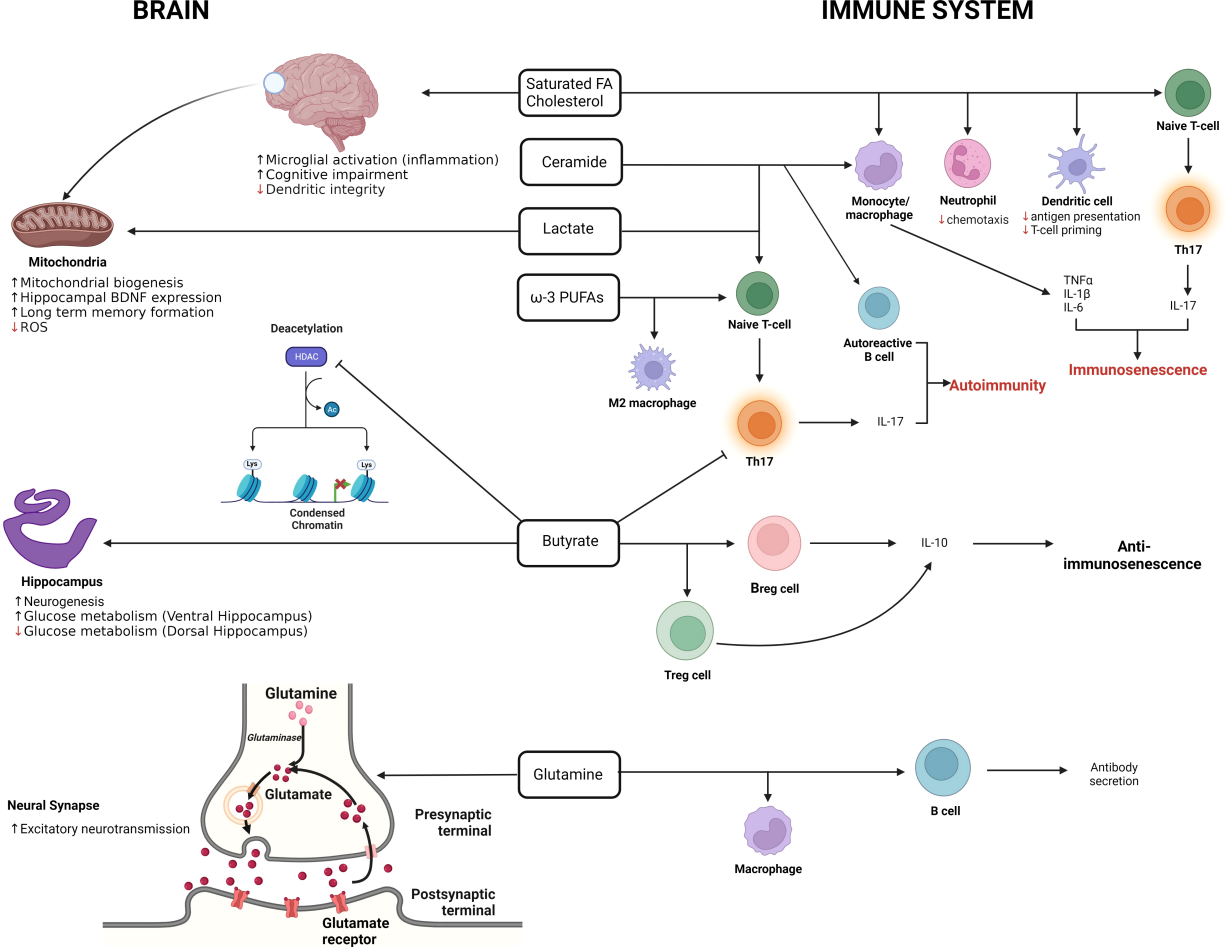
The double-edged role of metabolites in the brain and immune system. The illustration summarizes the effects of metabolites on the brain and immune system. In the immune system, metabolites such as SFAs, ceramides and lactate are promote the pro-inflammatory immunosenescent phenotype while butyrate promotes the anti-immunosenescent phenotype. In the brain, lactate, improves long term memory formation in the hippocampus, increases BDNF expression, promotes mitochondrial biogenesis and reduces ROS production. Butyrate on the other hand inhibits deacetylation and promotes neurogenesis in the brain. (Adapted from Conway *et.al.*, 2022 [143]).

Immunometabolic differences are bi-directional with different cellular activation and maturation states having specific metabolic requirements as well as the available nutrients and soluble factors influencing the immune state (reviewed in (144, 145). Indeed, there is an intense cross-talk between the immune system and the host metabolites and soluble factors that are present in the environment that the immune cells are exposed to. Many of these metabolites and soluble factors are derived from the host’s microbiome, which is also strongly affected by ELA (146, 147). These metabolites have significant immune-modulating potential (148), helping maintain the balance between immune tolerance and inflammatory phenotypes. In light of the ELA-induced immune senescence, it is interesting to note that several metabolite classes have been strongly associated with the induction of immunosenescence (**Figure 4**). The principal classes of metabolites associated with the induction of immunosenescence are saturated fatty acids, ceramides and lactate [**Figure 4** (148)].

Of these, differences in the metabolism of glutamine and arginine are concordant with the ELA immunophenotype. Glutamine is usually highly abundant and is essential for the proliferation of T cells (149), macrophages (150), as well as biasing macrophages towards the M1 and inflammatory phenotype cycle (150, 151), key elements of the ELA immunophenotype. Furthermore, the age-associated decline in glutamine levels has been hypothesised to “contribute towards the age-associated functional defects in immune cells” (148) that share many parallels with the ELA immunophenotype (34). Although glutamine doesn’t appear to be important in NK cells, arginine is essential for their proliferation and cytotoxicity, proliferation and cytokine secretion from T-cells (152, 153), and is a critical substrate for both arginase and the production of inducible NOS (iNOS). As arginine levels fall with age, it has been assumed to amplify age-related immunosuppression (16).

#### 4.4.1 Nutrient metabolism

As recently suggested, nutrient metabolism is very much an under-explored link between early life adversity, inflammation, and long-term disease risk (154). It is assumed that the immune system requires an adequate supply of both macro and micronutrients to develop correctly. However, there is now a growing literature that suggests that several different forms of psychosocial stress disturb nutrient uptake and metabolism, potentially associated with stress-induced alterations in the GI-microbiome, leading to altered development of the immune system (reviewed in (154, 155). Recently, Reid et al. suggested and subsequently demonstrated that iron deficiency may be an important factor, although, as our knowledge of alterations in ELA-induced microbiome and microbial metabolite changes grow, many other factors will become important (154, 156). Unfortunately, despite uncomplicated, cheap and effective treatment iron deficiency is perhaps the most common form of malnutrition affecting roughly 40% of those under 5 years old in the US (157), moreover, exposure to ELA is a significant risk factor for iron deficiency (156) and potentially in the development of the ELA immunophenotype (158). Like many other early life paradigms, iron deficiency in early life increases activity of pro-inflammatory cytokine induced gene expression pathways, reduces anti-inflammatory gene expression, and alters immune cell functional responses in mice. This could not be reversed despite the limited period of iron deficiency and the return to physiologically normal iron levels (158). Iron supplementation in adolescents, including in those with a prior history of ELA, was associated with decreased levels of inflammation (159).

Iron deficiency may not uniquely affect the ELA-associated immunophenotype. However, iron is essential in many post-natal developmental processes. Brain structure and function during this period also requires iron (156), as does synthesis of key neurotransmitters (dopamine, norepinephrine, and serotonin) (160) suggesting that it may play a role in other aspects of the overall ELA-phenotype.

#### 4.4.2 Mitochondrial metabolism and Intracellular stressors (ROS)

Mitochondrial metabolism is a critical component of the cells’ overall energy metabolism. Mitochondria serve as key signalling organelles in both the brain and immune system, in part through the production of metabolites such as cellular reactive oxygen species (ROS), which can modulate developmental pathways. ROS has long been thought of as a form of intra-cellular stress, as well as a key signalling molecule but mounting evidence suggests that it may also be a common intermediate of many psychosocial and early-life stressors. ROS production is induced, for example as part of the non-genomic actions of glucocorticoids (161) mediated by the membrane glucocorticoid receptor (92). Furthermore, long-term administration of GC down-regulates mitochondrial electron transport chain complex proteins, consequently increasing the production of ROS from the mitochondria (162, 163). This is not limited to exogenous GC administration, there is clear evidence for endogenous ROS in stress-related inflammatory (164), endocrine (165), as well as metabolic perturbations (166). Stem cells depend on anaerobic glycolysis for energy production in order to minimize the production of ROS and only shift toward mitochondrial oxidative phosphorylation (OXPHOS) during differentiation (167). Despite glycolysis generating less ATP compared to OXPHOS, its kinetics is faster and this enables it to reliably support rapid cell growth and proliferation of stem/progenitor cells. On the other hand, despite producing more ROS, OXPHOS is a more efficient way of generating ATP and is used in energy-demanding differentiated cells. One of the major features of senescence in cells is mitochondrial dysfunction coupled to the excessive build-up of intracellular ROS. Therefore, by minimising the amount of intracellular ROS, stem/progenitor cells are able to prevent senescence and they retain their proliferative capacity and ability to differentiate into different cell lineages. Interestingly, even after differentiation, hematopoietic cells still maintain the ability to switch between glycolysis and OXPHOS. For example, activated macrophages and dendritic cells (DCs) undergo a metabolic shift from mitochondrial OXPHOS towards glycolysis. These metabolic swifts have also been observed during neuronal cell differentiation and they are coupled to increases in mitochondrial biogenesis (168). From these data it is fundamentally evident that stem/progenitor cells have a dynamic energetic profile that is largely dependent on the stage of differentiation and that mitochondrial metabolism is a major player orchestrating the shift from glycolysis to OXPHOS. During exposure to ELA, there are complex intracellular processes taking place and among these, chronic exposure to GC increases mitochondrial ROS production, lowers ATP generation and inhibits mitochondrial biogenesis. While it may appear ambitious, the emergence of senescent immune cell phenotypes can be attributed to alterations in mitochondrial metabolism.

## 5 Moving away from brain-specific models to more holistic models of the effects of ELA

There is now a large body of observational clinical evidence that exposure to any number of stressors during this period is of critical importance in shaping an individual’s life trajectory of health and disease. Past studies on ELA have focused on the glucocorticoid receptor, HPA axis and fully differentiated somatic cells. Despite the considerable challenges and progress made, these have not provided us with convincing mechanisms behind the resultant phenotype and disease risk later in life. Here, we have looked at the role of concurrent programming of the immune and nervous systems in the long-term phenotype and trajectory induced by exposure to ELA to see if we can gain insight by comparing the two systems to find common underlying elements. It is now clear that these two systems act together to make a strong immune-brain axis that is disturbed by the developmental environment. A lot of effort has been made to understand what ELA actually is, with two competing hypotheses: cumulative risk, or dimensional models. The former is centred around the simple summation of the individual episodes of a wide range of types of adversity, whereas the latter is based on a more refined separation of the types of adversity into “dimensions of environmental experience that are shared across multiple forms of adversity” (169, 170). Unfortunately, the recent work separating adversity into component dimensions has had a clear neuro-developmental and psychopathological bias, and the underlying biological changes have been somewhat left aside. These dimensional models have proven highly successful in identifying the psychopathological effects of a threatening environment (emotional processing, reactivity and learning; increased threat sensitivity) that largely explains the preceding literature (170). Similarly, the axe “deprivation”, centred on paradigms including institutionalisation, neglect or reduced caretaker interactions largely explains the reduced social cognition, executive function and language deficiencies seen later in life (170–172).

**Figure 5:**
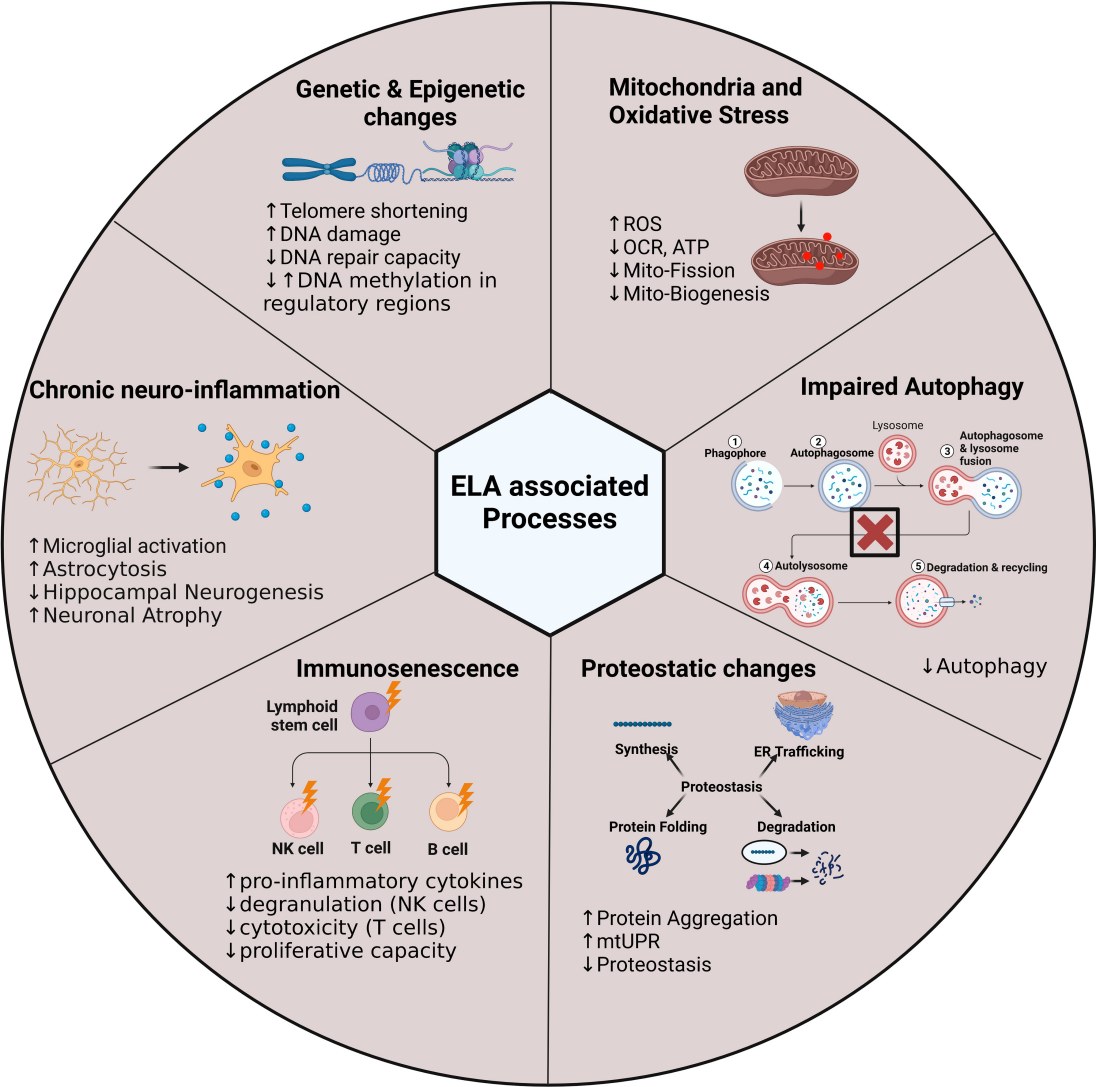
Early life adversity-associated processes in the brain and peripheral immune system. The illustration highlights the processes that we hypothesise to be altered in stem cells following exposure to ELA. These processes may harbour potential mechanisms to explain how the ELA phenotype is maintained over time. Adenosine Triphosphate, **ATP**; Deoxyribonucleic acid, **DNA**; Mitochondrial uncoupling protein response, **mtUPR**; Oxygen consumption rate, **OCR**; Reactive oxygen species, **ROS**.

The challenge is now to see how these dimensions can be transferred over to the disturbances seen in the immune system, and how the brain-immune axis is affected in each case. If, as we suspect, we will see a clear disturbance in the brain–immune homeostasis this will allow us to probe much further, and interrogate the biological mechanisms underlying the link not only between adversity and immune function, but to draw more biological and mechanistic conclusions behind the link to psychopathology. Over the past decades, the range of stressors and the later-life phenotypes and outcomes have expanded exponentially, however, despite several limited advances the underlying molecular mechanisms remain unclear. Indeed, for the majority of the observed phenotypes the affected tissues remain supposition, as do the intracellular processes affected (**Figure 5**). Similarly, the mechanism by which such phenotypes are maintained over many years or decades has been supposed to be epigenetic, however, this has never been definitively proven. The absence of such evidence led us to the current critical re-examination of the literature and the presentation of the “stem cell hypothesis” as an alternative potential mechanism.

When, as here, we take the immune system and brain together, it becomes clear, at the macroscopic level that common elements such as a lifelong turnover and production of new terminally differentiated cells offer an interesting explication for the long-term effects of ELA. Our previous data strongly suggests that early-life exposure to a stressor reduces the capacity of the immune system to generate subsequent generations of naïve cells, while others have shown that, early life stress impairs the capacity of neuronal stem cells to proliferate as they age. This leads us to the “stem cell hypothesis” whereby exposure to adversity during a sensitive period acts through a common mechanism in all the cell types by programming the tissue resident progenitor cells, thus ensuring a lifelong supply of functionally altered differentiated cells.

The “stem cell hypothesis” fits very nicely with the mechanistic differences seen in the differentiated cells we have investigated so far. One result stands out. Mitochondria do not only play a role in providing energy for cells, but, they play a key role in determining the fate of stem cells (particularly HSCs as outlined above). There is a lot of work currently ongoing as to how metabolites and metabolic process regulate differentiation and the fate of HSCs with unsuspected metabolites and signalling axes such as the non-classical retinoic acid pathway being implicated in controlling HSC destiny recently (167, 173). It does not take a great leap of imagination to imagine that ELA may alter mitochondria in the HSC and consequently alter the destiny of these cells, producing the lifelong “supply” of functionally altered differentiated cells. Indeed, the availability of anti-leukemic drugs targeting mitochondrial function e.g. Actinomycin D (174) may also open up avenues for exploring the role of mitochondrial dysfunction after ELA.

There are many challenges remaining. While the mechanisms in fully differentiated brain and immune cells remain poorly understood, it is perhaps time to look at the resident progenitor cells and what common mechanisms would produce functionally different immune and brain cells beyond the role for mitochondria that we have outlined here. We have outlined apparent flaws in our current approach to understanding the biological underpinnings of the Developmental Origins of Health and Disease. In past studies our approach extensively focussed on e.g. the glucocorticoid receptor, the HPA axis, and differentiated cells. However, a small number of emerging studies point to the correctness of our current assessment, and suggest that there are more fundamental and deep-rooted processes involved in the generation and maintenance of the overall ELA phenotype that may be common to all the tissues and systems affected.

## Data availability statement

The original contributions presented in the study are included in the article/supplementary material. Further inquiries can be directed to the corresponding author.

## Author contributions

Conceptualization: AM and JT; literature review: AM and JT; Manuscript writing and editing: AM and JT. Both authors contributed to the article and approved the submitted version.
